# Construction of Multiple Logic Circuits Based on Allosteric DNAzymes

**DOI:** 10.3390/biom12040495

**Published:** 2022-03-24

**Authors:** Xin Liu, Qiang Zhang, Xun Zhang, Yuan Liu, Yao Yao, Nikola Kasabov

**Affiliations:** 1 School of Computer Science and Technology, Dalian University of Technology, Dalian 116024, China; xinliuaisky@mail.dlut.edu.cn (X.L.); madao@mail.dlut.edu.cn (X.Z.); liuyuan.dlut@gmail.com (Y.L.); yy@mail.dlut.edu.cn (Y.Y.); 2 Knowledge Engineering and Discovery Research Institute, Auckland University of Technology, Auckland 1010, New Zealand; nkasabov@aut.ac.nz; 3 Intelligent Systems Research Center, Ulster University, Londonderry BT52 1SA, UK

**Keywords:** DNA computing, allosteric DNAzymes, logic circuits, threshold control, strand displacement

## Abstract

In DNA computing, the implementation of complex and stable logic operations in a universal system is a critical challenge. It is necessary to develop a system with complex logic functions based on a simple mechanism. Here, the strategy to control the secondary structure of assembled DNAzymes’ conserved domain is adopted to regulate the activity of DNAzymes and avoid the generation of four-way junctions, and makes it possible to implement basic logic gates and their cascade circuits in the same system. In addition, the purpose of threshold control achieved by the allosteric secondary structure implements a three-input DNA voter with one-vote veto function. The scalability of the system can be remarkably improved by adjusting the threshold to implement a DNA voter with 2n + 1 inputs. The proposed strategy provides a feasible idea for constructing more complex DNA circuits and a highly integrated computing system.

## 1. Introduction

DNA, the genetic material of all living organisms, can not only store biological genetic information, but also has powerful parallel computing capabilities, flexible programmability and predictable structure performance due to its Watson-Crick base pairing [1,2]. These traits make DNA an ideal nanomaterial, and it has been used in recent years to implement DNA nano-devices [3,4], DNA computing [5,6], DNA storage [7,8], drug delivery [9,10], and virus and ion detection [11,12,13]. In the field of computing, the advanced functions and complex operations of traditional electronic circuits can be integrated by adding appropriate logic gates and their cascades, but achieving the same functions as electronic circuits at the molecular scale is still difficult [14,15]. Fortunately, with the efforts of many scholars, diverse technologies provide great inspiration, and methods to implement DNA computing seem to be foreseeable [16,17,18]. Entropy-driven toehold mediated strand displacement reaction, a very mature technology, makes it simple to construct primitive logic gates [19]. Various endonucleases have been developed as an approach to regulate DNA strands and applied to construct a series of logic circuits [20,21]. Silver and copper nanoclusters templated by DNA strands have excellent fluorescence properties, and a variety of logic circuits have been implemented based on these properties [22,23,24]. G-quadruplex with horseradish peroxidation catalysis can replace traditional fluorescent labeling, which is employed as a reporter module for logical operations, making the system label-free [25,26]. The technology of DNA origami is utilized as a computing platform or carrier in the process of constructing logic circuits, or to fix the relative physical distance of DNA strands on its surface [27,28]. All of the technology mentioned above has a positive effect on DNA computing. However, it is still a challenge to be able to implement complex logic operations in a simple system.

Inspired by the powerful function of DNAzymes, which are catalytic nucleic acids with special DNA sequences, DNAzymes have the ability to catalyze the scission of ribophosphodiester linkage [29]. Isolation from random-sequence DNA libraries by “in vitro selection” is a general strategy for nearly all known DNAzymes [30]. Therefore, DNAzymes, similar to DNA molecules, possess many excellent properties compared to other strategies [21,22], including flexible design, simple synthesis and preservation. Meanwhile, it also has good regulation, programmability and efficient digestion, which has been proven to be a suitable tool for implementing DNA logic functions [29]. They have been widely utilized as processing modules for signal amplification and information delivery [31,32] and as molecular computing systems that implement a variety of logic gates and cascade functions [33,34]. In a typical DNAzyme-based digesting reaction, the activity of DNAzyme is a crucial factor to implement specific cleavage [30,35], and it relates to many factors including its own structure and the external environment, such as the structural integrity of the DNAzymes [36,37], the pH of the solution [38] and the types and concentrations of ions [39,40]. Therefore, under the premise of a suitable external environment, constructing a complete structure is the key point of implementing active cleavage for DNAzymes. Furthermore, DNAzymes are generally composed of conserved domains and recognition domains, and it is necessary to ensuring the invariance and integrity of the base sequence in the conserved domains to achieve the digestion effect. Thus, the activity of DNAzymes can also be adjusted by changes in the secondary structure of its conserved domain [41,42].

Different from the proposed strategies [36,43,44], we avoid the structure of four-way DNA junctions, the conformation of which is a mixture of three distinct species at low salt, while at higher concentrates of salt there still are two distinct species [45], which indicates that the purity of four-way junctions will be limited. Meanwhile, four-way DNA junctions are similar to three-way DNA junctions, both having a defined, non-flat geometry. The trigonal pyramidal shape of three-way junctions is more pronounced than the tetrahedral pyramidal geometry of four-way junctions, and the shape of a pyramidal makes a more compact conformation [46]. Therefore, the structure of three-way DNA junctions is preferable. In this study, the assembled DNAzyme is selected as the main processing module, the activity of which is regulated through controlling the assembly of DNAzyme as well as the conformational changes of the secondary structure of its conserved domain. To illustrate the feasibility of the system, some basic logic gates, such as AND, OR, XOR, and INH gates are implemented. In the construction of the basic modules, the allosteric structure of E6-type DNAzyme was modularly divided into two or three parts (MNAzyme) [37,47], which could assemble into an active DNAzyme. After the simple cascade of these basic logic gates, more complex operations, such as half-adder, half-subtractor, multiplexer (MUX) and demultiplexer (DEMUX) are successfully established to realize both arithmetic and non-arithmetic logic operations. Since the allosteric secondary structure of conserved domain can be used for threshold control, a three-input DNA voter with one-vote veto function is successfully implemented based on this allosteric strategy. In addition, to verify the powerful function of the threshold control, a DNA voter with more than three inputs can be achieved by increasing the threshold and the types of input, which further improved the scalability of the system. In the experiment part, native polyacrylamide gel electrophoresis (PAGE) and fluorescence measurement are utilized to verify the correctness of the results. The proposed allosteric DNAzyme based system with powerful flexibility provides a feasible idea for constructing highly integrated DNA circuits. It has a positive effect on simplifying the establishment of complex logic circuits, and may also have broad prospects in DNA computing, nano-devices, and information processing.

## 2. Materials and Methods

### 2.1. Materials and Chemical Reagents

All the DNA samples were purchased from Sangon Biotech. Co., Ltd. (Shanghai, China). Unmodified samples were purified by polyacrylamide gel electrophoresis (PAGE), and modified samples with fluorophore or RNA base were purified by high-performance liquid chromatography (HPLC). All DNA strands were stocked in deionized water (18.2 MΩ cm) and quantified by using Nanodrop 2000/2000c (Thermo Fisher Scientific Inc., Waltham, MA, USA). The sequences of all strands are listed in Table S1. Chemical reagents were purchased from Sangon Biotech. Co., Ltd. (Shanghai, China) and used without further purification.

### 2.2. DNA Assembly

In the INH logic gate and 2:1 MUX, D1_L_, D1_L_’ and D1_R_ were 10 pmol. D-F is 10.5 pmol, and the input DNA strands were both 10.5 pmol. The amount of each DNA strand contained in other logic gates and logic circuits was 20 pmol, the amount of input was 21 pmol, and the corresponding substrate was 40 pmol (TS_1_ and TS_2_) in the solution of 20 μL for PAGE analysis and 5 pmol (S_1_ and S_2_) in the solution of 100 μL for fluorescence assays, respectively. All complexes hybridized by two or more DNA strands were annealed using the same annealing procedure, and the single DNA strand required for logical operation can be added after the annealing. DNA strands were annealed in the incubation buffer (10 mM Tris-HCl, 20 mM acetate acid, 1 mM EDTA, 500 mM NaCl, 100 mM MgCl_2_·6H_2_O, pH = 7.8), heated to 90 °C for 5 min, steadily cooled to 85 °C for 5 min, and then steadily cooled to 25 °C for 2 h, finally kept at room temperature for the next operation. Input DNA strands were heated to 90 °C for 10 min and cooled to room temperature before being added to the solution.

### 2.3. Native PAGE

All the DNA strands were added into the solution of 20 μL as required in the DNA assembly section, and the 60% glycerol of 3 μL was added after the 3 h incubation time. Then, the solution was analyzed on 12% native polyacrylamide gel in 1 × TBE/Mg^2+^ buffer consisting of Tris base (89 mM, pH = 7.8), boric acid (89 mM), EDTA (2 mM) and MgCl_2_·6H_2_O (100 mM). The gels were run on a DYY-6D electrophoresis apparatus (Beijing Liuyi Co., Ltd., Beijing, China) at a constant voltage of 80 V for 3 h.

### 2.4. Measurement of Fluorescence Spectroscopy

The mixture of Tris-HCl (10 mM, pH = 7.8), acetate acid (20 mM), EDTA (1 mM), NaCl (500 mM), MgCl_2_·6H_2_O (100 mM) and KCl (4 mM) was used as the incubation buffer for the fluorescence assays. All the DNA strands were added to the solution of 100 μL. The excitation and emission wavelengths of ROX were 578 nm and 604 nm, and the excitation and emission wavelengths of FAM were 492 nm and 518 nm, respectively. All the fluorescence assays were implemented using TECAN Microplate Reader Spark 20M (Tecan Trading Co., Grödig Australia). The fluorescence intensities were recorded every 10 min.

## 3. Results

### 3.1. Mechanism of the Allosteric Strategy and DNAzyme Assemblying

#### 3.1.1. Realization of the INH Gate

**Figure 1:**
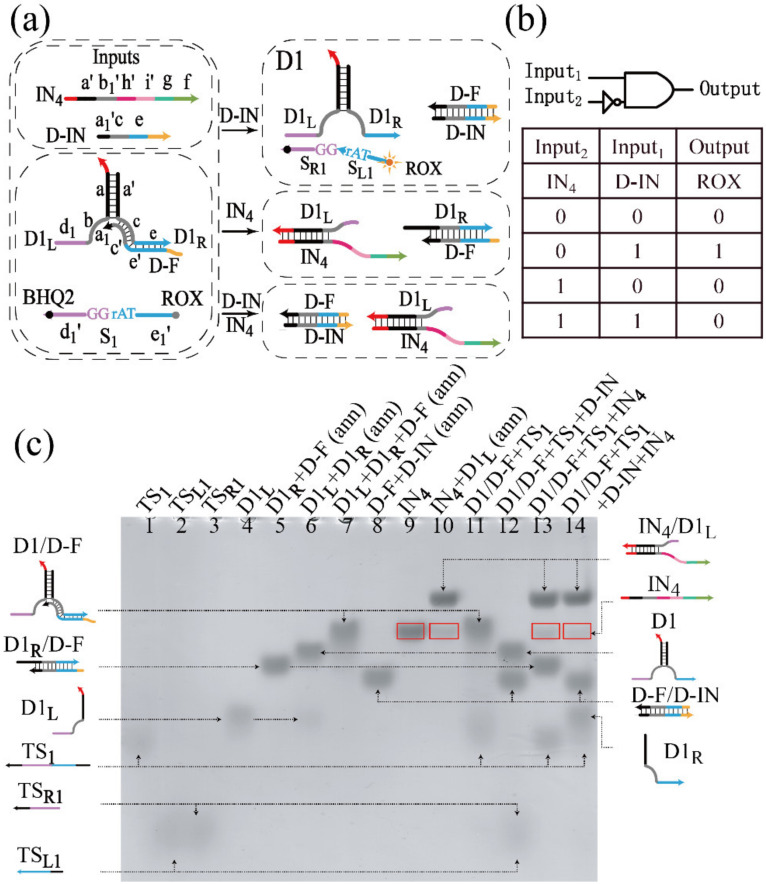
(**a**) Schematic diagram of the INH gate. (**b**) Truth table and logical operations of INH gate. (**c**) PAGE gel of the INH gate. The DNA strands and operations are marked above the lane number. Lane 1, substrate TS_1_; lane 2, DNA strand TS_L1_; lane 3, DNA strand TS_R1_; lane 4, DNA strand D1_L_; lane 5, the annealing of the mixture of D1_L_ and D-F; lane 6, annealing operations for the mixture of D1_L_ and D1_R_; lane 7, annealing operations for the mixture of D1_L_, D1_R_ and D-F; lane 8, annealing operations for the mixture of D-F and D-IN; lane 9, DNA strand IN_4_; lane 10, annealing operations for the mixture of IN_4_ and D1_L_; lane 11, the initial state of the INH gate: substrate strand TS_1_ and the annealing mixture of D1_L_, D1_R_ and D-F; lane 12, the initial state of the INH gate with the addition of Input_1_ (D-IN); lane 13, the initial state of the INH gate with the addition of Input_2_ (IN_4_); lane 14, the initial state of the INH gate with the addition of Input_1_ (D-IN) and Input_2_ (IN_4_). All the solutions are incubated at 21 °C for 3 h. Slashes linking is used to represent the DNA complexes.

To illustrate the principle of allosteric strategy, some basic logic gates were constructed. Taking the Inhibit gate as example, the Inhibit logic is shown in Figure 1a. The DNAzyme D1 was assembled through the hybridization between the domains a and a’ in two oligonucleotide strands D1_L_ and D1_R_. D1_L_ had the split conserved domain b and the half recognition domain d_1_. D1_R_ had the other half domains, c and e. After D1_L_ mixed with D1_R_, domains, b and c could form the complete conserved domain. In addition, D-F containing c’ and e’, the complementary domains to c and e of strand D1_R_, could inhibit the activation of D1. Therefore, in the initial state of the INH gate, the partially conserved domain of D1 was shielded by the secondary structure hybridized with D-F. Then, D1 was inactive and could not digest the corresponding substrate. After inputting DNA strand D-IN, which had the ability to displace D1_R_ from D-F, D1 was activated and could break the ribose phosphodiester bond of the corresponding substrate S_1_ at the ‘TrAGG’ site. Here, the ROX fluorescence was labeled at the 5′-terminal of S_1_, quenched by BHQ2 modified at the 3′-terminal of S_1_. D1_R_ was displaced from D1_L_ with the addition of IN_4_, resulting in the destruction of the complete structure of D1, even if D-IN existed in the solution. The truth table and logical operations of the INH gate are shown in Figure 1b. The presence or absence of input was defined as “1” or “0”, which was available for all the logic circuits in this work.

A PAGE experiment was conducted to verify the INH gate, as shown in Figure 1c. In order to distinguish the short S_L1_ and S_R1_ produced by the cleavage of S_1_, six unrelated T bases were added to the 3′ and 5′-terminals of S_1_, which was renamed TS_1_, and the length-changed strands S_L1_ and S_R1_ were renamed TS_L1_ and TS_R1_, respectively. The stable existence of complexes in the solution was verified, including the complex of triple-stranded D1/D-F and the substrate TS_2_. From Figure 1c, the two bands of D1/D-F and TS_2_ can be clearly observed in lane 11, and their structures were stable with no addition of inputs. With the addition of D-IN, the structure of D1/D-F was destroyed, and the activated D1 was produced in this process, which had the ability to digest TS_1_. Thus, TS_R1_ and TS_L1_ produced by the cleavage of TS_1_ and the double-stranded D1 appeared in lane 12. With the addition of IN_4_, IN_4_/D1_L_ was produced in the solution, and the concentration of D1/D-F was reduced in the process. Therefore, the bands of IN_4_/D1_L_ and D1_R_/D-F appeared in lane 13, which verified the rationality of the schematic. It can be found that the band marked by the red box appeared in the gel. By comparing lanes 9 and 10, it can be derived that the red box in lane 10 was IN_4_. In lane 13, since the amount of IN_4_ was more than D1_L_ in solution, the excessive IN_4_ can be observed in the gel, and the same phenomenon occurred in lane 14. Meanwhile, the leakage can be sensitively detected by fluorescence experiments in curve 4 of Figure S1. From the analysis of the reaction process, most of D1 were disassembled with the addition of two inputs, but there was still activated D1 resulting in the leakage. The activated D1 was produced by the hybridization of D-IN and D-F, while there was no hybridization between D1_L_ and IN_4_. Therefore, the band of TS_1_ in lane 14 was grey, and the band of output cannot be observed, which was the shortcoming of PAGE experiment.

#### 3.1.2. Realization of the OR Gate

**Figure 2:**
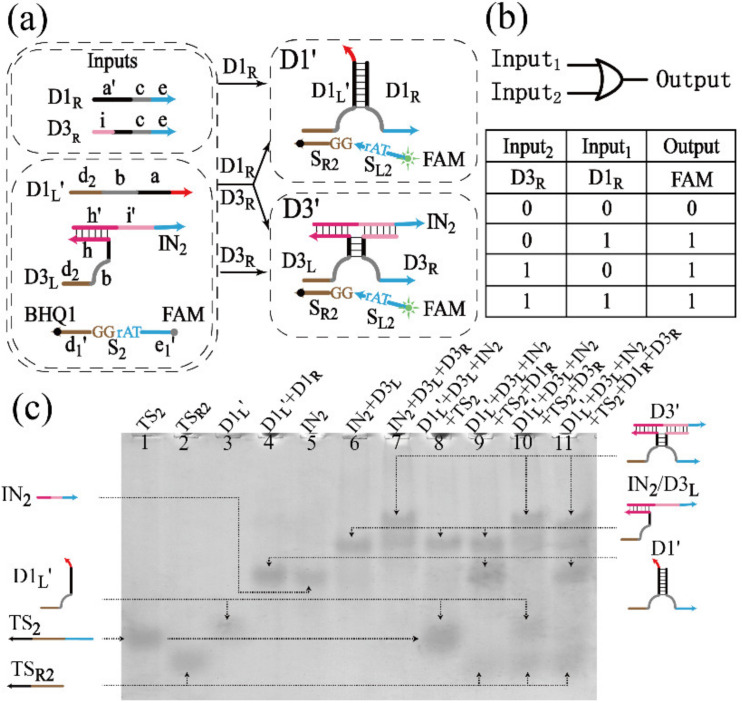
(**a**) Schematic diagram of the OR gate. (**b**) Truth table and logical operations of the OR gate. (**c**) PAGE gel of the OR gate. Lane 1, substrate TS_2_; lane 2, DNA strand TS_R2_; lane 3, DNA strand D1_L_’; lane 4, the annealing of the mixture of D1_L_’ and D1_R_; lane 5, DNA strand IN_2_; lane 6, the mixture of IN_2_ and D3_R_; lane 7, the mixture of IN_2_, D3_L_ and D3_R_; lane 8, the initial state of OR gate: the mixture of D1_L_’, D3_L_, IN_2_ and TS_2_; lane 9, the initial state of OR gate with the addition of Input_1_ (D1_R_); lane 10, the initial state of OR gate with the addition of Input_2_ (D3_R_); lane 11, the initial state of OR gate with the addition of Input_1_ (D1_R_) and Input_2_ (D3_R_). All the solutions are incubated at 21 °C for 3 h.

Since the domain in one DNA strand can be easily replaced by any other domain based on the modular design of DNA strands, the domain d_1_ in D1_L_ was replaced by d_2_, which was renamed D1_L_’, and the DNAzyme assembled by the hybridization between D1_L_’ and D1_R_ was named D1′, which could digest substrate S_2_. The FAM fluorescence was labeled at the 5′-terminal of S_2_, quenched by BHQ1 modified at the 3′-terminal of S_2_. In Figure 2a, DNAzyme D3′ was divided into three parts, and the OR logic gate was implemented by assembled D1′ and D3′. The process to assemble D1′ would be promoted by D1_R_, similarly, D3′ would be assembled with the addition of D3_R_. The truth table and logical operations of the OR gate are shown in Figure 2b.

The PAGE experiment was conducted to verify the OR gate, as shown in Figure 2c. In order to obtain the clear band of the output produced by the digestion of S_2_, twelve unrelated T bases were added to the 3′-terminal of S_2_, which was then named TS_2_, the longer products of which was named TS_R2_. The band of TS_R2_ could be observed in lanes 9–11, and the DNAzyme D1′ (D3′) was successfully assembled in lanes 9 and 11 (lanes 10 and 11). It can be found that the hybridization by three DNA strands was inadequate, always accompanied by the generation of double-stranded DNA (the bands similar to IN_2_/D3_R_ in lanes 7, 10 and 11 of Figure 2c), and the unknown band was not labeled. According to Figure S5b, since the bases of IN_2_/D3_L_ and IN_2_/D3_R_ were basically the same, their bands were run to the same position (lanes 7 and 8). An extra band was produced after the hybridization of IN_2_, D3_L_ and D3_R_ (lanes 9 and 10), which was at the same position as IN_2_/D3_L_ or IN_2_/D3_R_. Therefore, the unknown band in Figure 2c may be IN_2_/D3_L_, IN_2_/D3_R_ or their mixture, which resulted in the decrease in the amount of activated D3′, and it can also explain the phenomenon that curve 3 was slower than curve 2 in Figure S2. In addition, the same principle was also adopted to construct the AND gate and the XOR gate, the schematic diagrams, PAGE analysis and fluorescence assays of which are shown in Figures S3 and S4. Based on these basic logic gates, more complex and advanced circuits were constructed.

**Figure 3:**
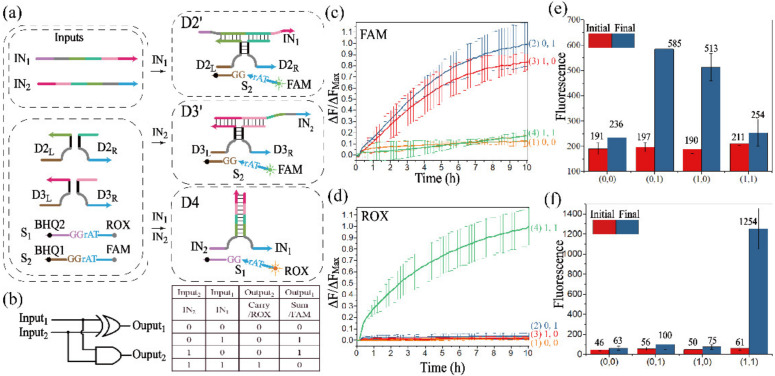
(**a**) Schematic diagram of the half-adder. (**b**) Truth table and logical operations of the half-adder. Signals of SUM bit (**c**) and CARRY bit (**d**) represented by the time-dependent normalized fluorescence curves of FAM and ROX. The fluorescence intensities are recorded every 10 min. (1) No input is added; (2) IN_1_ is added; (3) IN_2_ is added; (4) IN_1_ and IN_2_ are both added. The real fluorescence of SUM bit (**e**) and CARRY bit. (**f**) The initial state: red columns, and the final state: blue columns. All data represent the average of three replicates. Error bars represent one standard deviation from triplicate analyses, which is available for all the fluorescence assays of logic circuits in this work.

However, we found that the stable work of DNAzymes was the basis of the construction of computing systems, and the temperature had a significant effect on the activity of assembled DNAzymes. There were only two different structures of DNAzyme applied in this work, which were double-stranded and three-way junction, respectively. The double-stranded DNAzymes were D1, D1′ and D4 (Figure 1a). The hybridized parts of D1 and D1′ were the same, but D4 had the longest double-stranded part (Figure S6d). The three-way junctions were D2, D3, D2′ and D3′ (Figures S4a and Figure 3a), and their quantity of bases in the hybridized region were the same. Therefore, D1′, D2 and D4 were selected to explore the reaction rates under different temperatures, as shown in Figure S6. According to these experimental results, 21 °C was selected as the appropriate temperature for all of the following operations.

### 3.2. Implrmentation of Arithmetic Functions

#### 3.2.1. Realization of the Half-Adder

Both the half-adder and the half-subtractor are important logic circuits for arithmetic functions. A half-adder performs binary addition operations of (0 + 0), (0 + 1), (1 + 0) and (1 + 1), which are implemented by mapping two inputs to the combined outputs of the SUM bit and the CARRY bit. In order to distinguish the output signals of these two bits, a parallel DNAzyme processing module needed to be constructed to identify substrates S_1_ and S_2_.

Figure 3a outlines the schematic diagram of the half-adder. Three assembled DNAzymes were utilized to implement the half-adder logic operation. DNAzymes cannot be assembled with no addition of inputs, and the substrate cannot be digested. D2′ (D3′) was assembled by the input strand IN_1_ (IN_2_), leading to a significant rise of FAM fluorescence. With the addition of two inputs, the stronger affinity between IN_1_ and IN_2_ would promote the assembly of D4, recognizing and digesting substrate S_1_, and the fluorescence of ROX was released. Therefore, the assembly of DNAzymes D2′ and D3′ implemented the function of the XOR gate (Figure S4), the output of which corresponded to the CARRY bit. The AND gate (Figure S3) was implemented by assembling D4, the output of which corresponded to the SUM bit. The truth table and logical operations of the half-adder are shown in Figure 3b.

Since S_1_ and S_2_ were labeled with ROX and FAM, respectively, fluorescence assays were conducted to monitor the different signals of half-adder, as shown in Figure 3c,d. Here, if the normalized fluorescence intensity was lower than 0.3 or higher than 0.7, the output would be defined as “0” or “1”. Curve 1 in Figure 3c,d were Output_1_ and Output_2_, respectively, and remained negative without any addition of input, which realized the operation of (0 + 0 = 0 0). In the presence of IN_1_ or IN_2_, the signals of curves 2 and 3 in Figure 3c had a significant increase, and their corresponding fluorescence of ROX remained negative in Figure 3d, confirming the successful assembly of D2′ or D3′, and the operations of (0 + 1 = 0 1) or (1 + 0 = 0 1) were implemented. When IN_1_ and IN_2_ co-existed, curve 4 significantly increased in Figure 3d, and its normalized fluorescence intensity of FAM was negative in Figure 3c. The initial and final fluorescence value during the reaction are shown in Figure 3e,f. These results demonstrated the successful construction of the half-adder.

Moreover, the operations of the half-adder were verified by the PAGE experiment (Figure S7), and the results further confirmed the construction of the half-adder. Furthermore, D2′ and D3′ could be assembled at room temperature, which was verified by the PAGE analysis (Figure S5b).

#### 3.2.2. Realization of the Half-Subtractor

**Figure 4:**
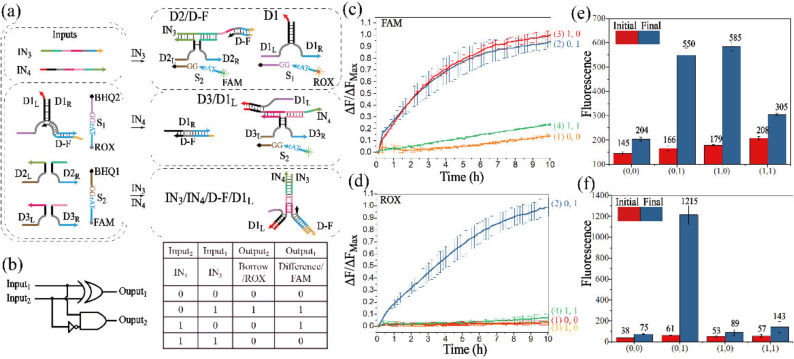
(**a**) Schematic diagram of the half-subtractor. (**b**) Truth table and logical operations of the half-subtractor. Time-dependent normalized fluorescence curves of the DIFFERENCE bit (**c**) and the BORROW bit (**d**). The fluorescence intensities are recorded every 10 min. (1) No input is added; (2) IN_3_ is added; (3) IN_4_ is added; (4) IN_3_ and IN_4_ are both added. The real fluorescence of the difference bit (**e**) and the borrow bit (**f**). The initial state: red columns, and the final state: blue columns.

A half-subtractor performs binary subtraction operations of (0 − 0), (0 − 1), (1 − 0) and (1 − 1), which are implemented by mapping two inputs to the combined outputs of the DIFFERENCE bit and the BORROW bit. The operation of the half-subtractor is illustrated in Figure 4a. IN_3,_ with the complementary domains to DNA strand D-F, had the ability to displace strand D1_R_ from strand D-F, which activated D1. Another input, IN_4,_ could displace D1_R_ from D1_L_, which prevented the assembly of D1. However, IN_3_ and IN_4_ preferentially hybridized with each other when they co-existed. Under this condition, D2 and D3 cannot be assembled, and the activity of D1 was suppressed by strand IN_4_. The truth table and logical operations of the half-subtractor are shown in Figure 4b.

The fluorescence curves with error bar of FAM and ROX were plotted against real time to monitor the half-subtractor, as shown in Figure 4c,d. From Figure 4c, the fluorescence of curve 1 was negative during the whole reaction, which was consistent in Figure 4d, corresponding to the operation of (0 − 0 = 0 0). The operation of (0 − 1) was transferred to (2 − 1) by a high borrow bit. In curve 2 of Figure 4c,d, the fluorescence increased with the addition of IN_3_, and the BORROW and DIFFERENCE bits were both endowed as “1”. In the presence of IN_4_, curve 3 had a significant increase in Figure 4c, but its fluorescence signal stayed at a low intensity in Figure 4d, corresponding to the operation of (1 − 0 = 0 1). Finally, the operation of (1 − 1 = 0 0) was monitored by curve 4 in Figure 4c,d. The initial and final fluorescence values are shown in Figure 4e,f.

In addition, the half-subtractor was further verified by PAGE analysis (Figure S8), and the DNA complexes such as D2/D-F, D1′ and D3/D1_L_’ could be observed. The half-subtractor logical circuit was further confirmed by these results.

### 3.3. Implementation of Non-Arithmetic Functions

#### 3.3.1. Realization of the 2:1 MUX

**Figure 5:**
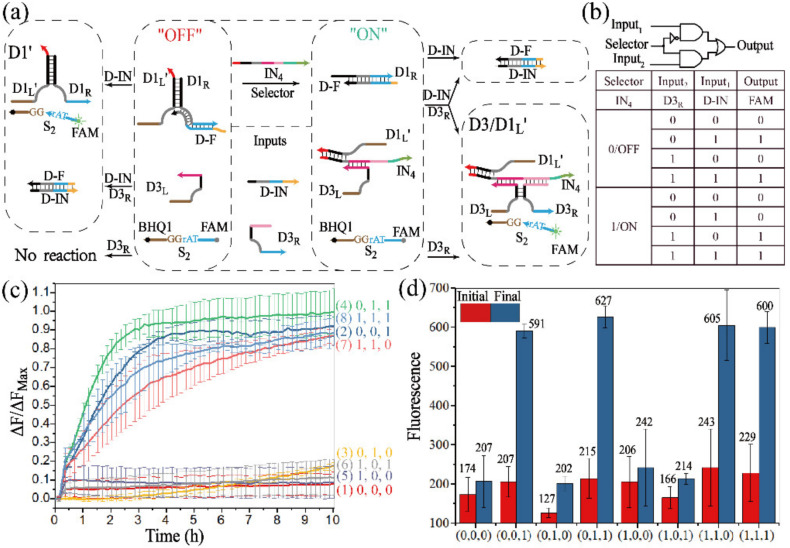
(**a**) Schematic diagram of the 2:1 MUX. (**b**) Truth table and logical operations of the 2:1 MUX. (**c**) Time-dependent normalized fluorescence curves. The fluorescence intensities are recorded every 10 min. “OFF” state: the selector IN_4_ is absent, (1) no input is added; (2) D-IN is added; (3) D3_R_ is added; (4) D-IN and D3_R_ are both added. “ON” state: the selector IN_4_ exists, (5) no input is added; (6) D-IN is added; (7) D3_R_ is added; (8) D-IN and D3_R_ are both added. (**d**) The real fluorescence changes of 2:1 MUX. The initial state: red columns, and the final state: blue columns.

Arithmetic logic can be implemented by the half-adder and half-subtractor, but non-arithmetic operations are also required in molecular computing. In the non-arithmetic aspect, a MUX can transmit multiple inputs into a single output channel during data transmission, which corresponds to data compression, and a DMUX can operate the opposite function. The operation of the 2:1 MUX is illustrated in Figure 5a. Since one of the characteristics of the MUX is the single output channel, the different assembled DNAzymes should digest the same substrate. To achieve this function, the DNAzyme D1′ was designed to recognize and digest the same substrate S_2_ as DNAzyme D3. In the operation of the 2:1MUX, the signal of two input streams was selectively transmitted into a single output channel by the “ON” (IN_4_ was present) or “OFF” state (IN_4_ was absent). Only D1′ could be activated in the “OFF” state with the addition of strand D-IN, and only D3 could be activated in the “ON” state by the addition of strand D3_R_. The truth table and logical circuit of 2:1 MUX are shown in Figure 5b.

The operations of 2:1 MUX were confirmed by fluorescence assay, as shown in Figure 5c. Curves 1–4 corresponded to the “OFF” state. When D-IN was added, the fluorescence intensity had a great increase (curves 2 and 4), while curves 5–8 corresponded to the “ON” state, and the fluorescence intensity significantly increased once D3_R_ was added (curves 7 and 8), which was consistent with the truth table. The comparison of the fluorescence intensities of the initial and the final states is shown in the Figure 5d. In addition, to further verify the operations of the 2:1 MUX, the gel electrophoresis experiment was carried out (Figure S9).

#### 3.3.2. Realization of the 1:2 DEMUX

**Figure 6:**
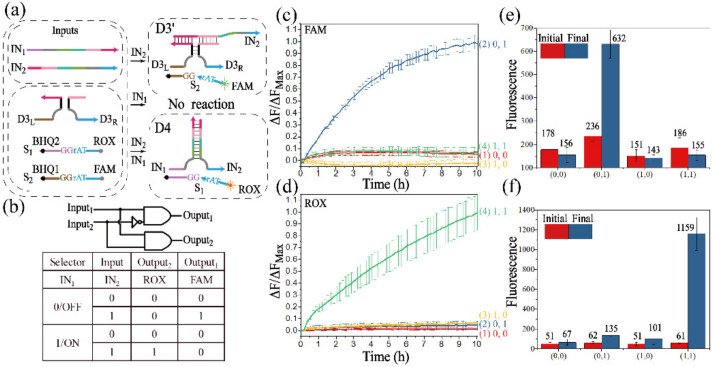
(**a**) Schematic diagram of the 1:2 DEMUX. (**b**) Truth table and logical operations of the 1:2 DEMUX. Time-dependent normalized fluorescence curves of Output_1_ (**c**) and Output_2_ (**d**). The fluorescence intensities are recorded every 10 min. “OFF” state: IN_1_ is absent, (1) IN_2_ is absent; (2) IN_2_ is added. “ON” state: IN_1_ exists, (3) IN_2_ is absent; (4) IN_2_ is added. The fluorescence changes of FAM (**e**) and ROX (**f**). The initial state: red columns, and the final state: blue columns.

The XOR gate (Figure S4a) is actually a parallel connection of two INH gates with the same output, and the INH gate can be successfully constructed by part of the XOR gate. Unlike the approach proposed in Figure 1a, DNAzyme D3′ was utilized to implement the INH function, the inputs of which were also adopted to assemble D4, and the AND function was implemented. With the establishment of the basic modules, the 1:2 DEMUX could easily be constructed by the parallel connection of an INH gate and an AND gate. As shown in Figure 6a, D3′, which was capable of identifying and digesting substrate S_2_, could be successfully assembled in the presence of IN_2_, but invalidated with the addition of IN_1_. In contrast, D4, which was capable of identifying and digesting substrate S_1_, would be activated by the co-existence of IN_1_ and IN_2_. The truth table and logical operations of 1:2 DEMUX are shown in Figure 6b.

The fluorescence curves of Output_1_ and Output_2_ are depicted in Figure 6c,d, respectively. It could be observed that, in the presence of IN_2_, a significant fluorescence of FAM was produced in curve 2 of Figure 6c, but the fluorescence of ROX was basically negative in curve 2 of Figure 6d. The fluorescence of ROX remarkably increased in curve 4 of Figure 6d with the addition of the IN_1_ and IN_2_, and the corresponding fluorescence of FAM was negative in curve 4 of Figure 6c. No significant increase of fluorescence could be observed when no input or only IN_1_ was added. The results of a fluorescence assay were consistent with the truth table. The initial (the red column) and final fluorescence value (the blue column) of Output1 and Output2 are shown in Figure 6e,f. In addition, the 1:2 DEMUX circuit was further verified by PAGE analysis (Figure S10), which further confirmed the construction of 1:2 DEMUX.

### 3.4. Realization of Threshold Control and DNA Voter with One-Vote Veto Function

For a universal system, it is critical to perform diverse functions. Majority logic gates can be used in fault-tolerant computing or as construction blocks to implement advanced logic circuits [48]. A majority logic gate will have true outputs if more than half of the inputs are true, which works as a voter that only passes by more than half of the votes. Besides, the DNA voter possesses the function to model the well-known voting mechanism of the United Nations Security Council, in which a permanent member can reject any proposal via the right of “One-vote veto [49]”. We found that the allosteric secondary structure conformation of its conserved domain could be used for threshold control. The allosteric secondary structure conformation was also applied in INH, half-subtractor and 2:1 MUX. In these processes, D-F hybridized with D1_R_ in advance to form the double strand to ensure that there was no free D1_R_ in the solution, which can be achieved by the annealing operation, and the ratio between D-F and D1_R_ was set as 1.05:1. In the construction of the DNA voter, DNAzymes were catalytic nucleic acids, so, non-DNA substances would not be added to the system as input.

**Figure 7:**
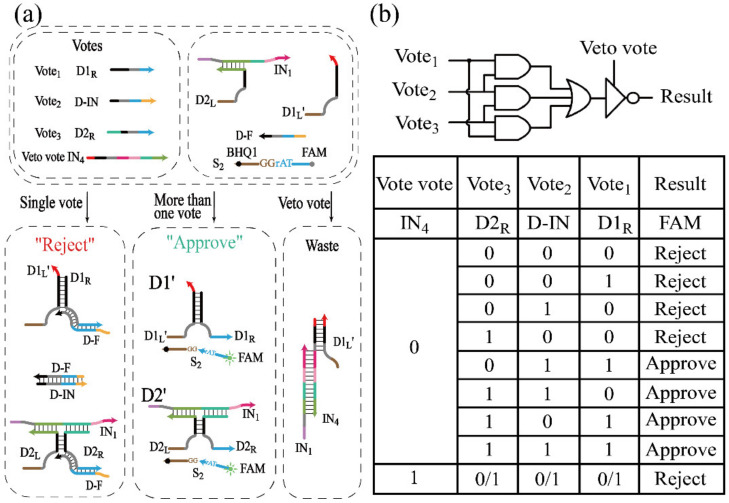
(**a**) Schematic diagram of the DNA voter with the one-vote veto function. (**b**) Truth table and logical operations of the DNA voter.

As mentioned, a DNA voter with a one-vote veto function was successfully established by the strategy shown in Figure 7a, in which DNAzymes D1′ and D2′ were utilized to implement the majority logic gate, and they could recognize and digest the same substrate S_2_. Herein, the DNA strands D1_R_, D-IN and D2_R_ functioned as three inputs. DNA strands D1_R_ and D2_R_ were inputted to assemble the DNAzymes D1′ and D2′, respectively. The allosteric secondary structure conformation was used in the INH (Figure 1), the half-subtractor (Figure 4), and the 2:1 MUX (Figure 5) devices. In these logic operations, D-F hybridized with D1_R_ in advance to form a double strand to ensure that there was no free D1_R_ in the solution, which can be achieved by the annealing operation, and the ratio between D-F and D1_R_ was set as 1.05:1. However, different from these devices, D-F was free in the solution in the initial state of the DNA voter (Figure 7a), and the inputs would hybridize with other strands besides D-F without the premise of the annealing operation. Since the hybridization reaction between the inputs and D-F would be accelerated with the increase of the concentration of D-F, in the construction of the DNA voter, DNA strand D-F, the concentration of which was as the threshold, had the ability of changing the secondary structure of the conserved domain to inactivate D1′ and D2′. Although the DNA strand D-IN could not be used to assemble any DNAzymes, it had the priority over the other two inputs to consume the amount of D-F that was completely complementary to strand D-IN (Figure S11d,e). Hence, D-F would not be completely consumed when only one input existed in the solution; once two or more inputs were introduced in the solution, the DNA strand D-F would be completely consumed, and D1′ or D2′ would be assembled by the rest of the inputs. The detail of the hybridization principle between inputs and D-F and the design of the toehold in each strand were studied in Figure S11a.

To implement the one-vote veto function, IN_4_ was introduced as the veto input to prohibit the assembly of DNAzymes, which had the highest priority over the other inputs. DNA strand IN_4_ could hybridize with DNA strands D1_L_ and IN_1_ to prevent the assembly of D1′ and D2′ even if any inputs existed in the solution. The truth table and logical operations of the DNA voter are shown in Figure 7b.

**Figure 8:**
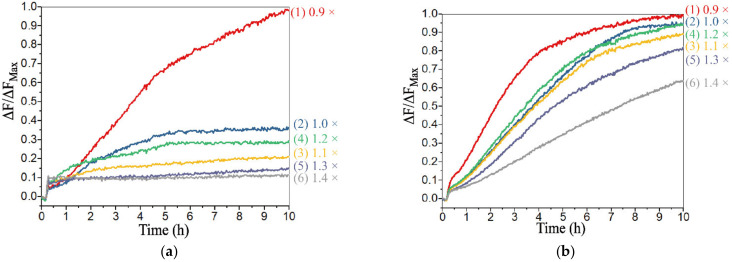
Fluorescence analysis for different concentrations of DNA strand D-F, which are 0.9×, 1.0×, 1.1×, 1.2×, 1.3× and 1.4 × 0.5 μM, respectively. The fluorescence intensities are recorded every 10 min. (**a**) DNA strand D1_R_ is added to the voter. (**b**) DNA strands D-IN and D2_R_ are added to the voter.

However, in the construction of the voter, the system would be abnormal with an extreme concentration of D-F. In order to ensure that there would be no leakage with the addition of any sole input, and that the reaction rate would not be too slow with the addition of any two inputs, six groups of different concentration of D-F were introduced, which were 0.9×, 1.0×, 1.1×, 1.2×, 1.3× and 1.4 × 0.5 μM, respectively. Since the reaction could not occur with only the presence of D-IN, the process with the addition of D1_R_ was explored. As shown in Figure 8a, only D1_R_ was added, and it can be found that the leakage of the reaction was obvious when the concentration of D-F was less than 1.0×. However, the leakage could be effectively limited to 0.3 or less when the concentration of D-F was greater than 1.1×. When D2_R_ was added, the reaction rate and the leakage were significantly reduced (Figure S12a). The addition of D-IN and D2_R_ were explored in Figure 8b. The reaction rate was slow when the concentration was greater than 1.4×. The addition of D-IN and D1_R_ (D1_R_ and D2_R_) was explored in Figure S12b (Figure S12c), and it was clear that all the outputs reached a positive state during the reaction process. Therefore, from the above results, the appropriate concentration range of D-F was 1.1–1.3×, and 1.2× was selected as the optimal concentration to obtain the best inhibition. In addition, the system was scalable based on the threshold controlled allosteric DNAzymes. The voting device designed in this study has two output states, which correspond the “Approve” and “Reject”. However, when a voter with 2n inputs is used to vote, it may happen that the affirmative vote is the same as the negative vote, and the output results cannot be matched. In theory, a DNA voter of more than three inputs can be achieved by increasing the threshold and adding the types of input (a DNA voter with 2n + 1 (n = 1, 2, 3…) inputs was designed in Figure S14), which remarkably improved the scalability of the system.

**Figure 9:**
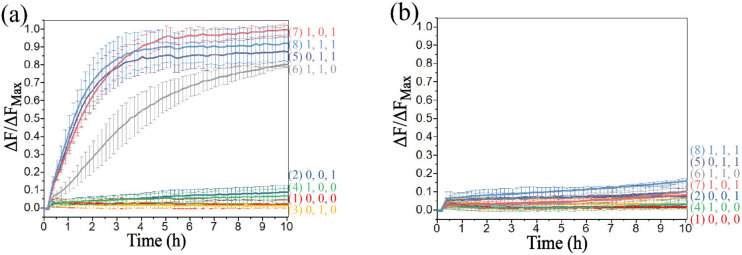
Time-dependent normalized fluorescence curves without (**a**) and with (**b**) the addition of IN_4_. The fluorescence intensities are recorded every 10 min. (1) No input is added; (2) D1_R_ is added; (3) D-IN is added; (4) D2_R_ is added; (5) D1_R_ and D-IN are added; (6) D2_R_ and D-IN are added; (7) D1_R_ and D2_R_ are added; (8) D1_R_, D-IN and D2_R_ are added.

The fluorescence curves of the voter without the addition of IN_4_ are plotted in Figure 9a. In the case of a vote not passed, there was no fluorescent variation in curves 1–4 corresponding to no input or the addition of single input to the solution. In the case of a passed vote, the fluorescence curves 5–8 had a remarkable improvement with the addition of more than half of the inputs. The fluorescence curves corresponding to the addition of the veto vote IN_4_ are shown in Figure 9b. It was clear that all votes were rejected once IN_4_ was added. The initial (the red column) and final fluorescence value (the blue column) of the results are shown in Figure S13. Overall, these results verified the DNA voter with the one-vote veto function.

## 4. Discussion

In the field of DNA computing, achieving the same functions as electronic circuits at the molecular scale is still difficult. To this extent, in the present study we aimed to construct a powerful system with multiple logical operations. DNAzymes were utilized to construct basic logic gates, and a voter with a one-vote veto function was further implemented based on the strategy of threshold regulation. However, there were still challenges in the construction of large scale cascade circuits, including the leakage and efficiency in reactions, which are common problems in DNA computing [19,21,33].

The process of leakage can be suppressed by rational DNA sequence design, but the influence of leakage cannot be eliminated, which is inevitable in biochemical reactions. Here, the associated experimental strategies and operations are optimized to avoid the structures with the possibility of leakage. For example, in the construction of INH gate (Figure 1a), to obtain the complex hybridized by D1_R_ and D-F without impurities, their ratio was preferably set as 1:1. But in the actual operation process, the amount of D-F and D1_R_ cannot be exactly the same. Although the excessive D-F could not activate D1, the excessive D1_R_ could hybridize with D1_L_ to generate activated D1, resulting in the leakage. Therefore, D-F was required to be slightly excess to D1_R_, and the concentration ratio between D-F and D1_R_ was set as 1.05:1.

For the above operations, the process to manipulate the ratio between DNA strands could be avoided by the design of the hairpin structure [42]. However, the function of threshold control cannot be implemented, and it is unfavorable for the construction of the DNA voter. Additionally, the strategy of changing the conformation of DNAzymes by allosteric regulation has also been used to alter the activity of DNAzymes [41]. With this strategy, the exposed recognition domain of the DNAzyme could be produced, which could directly hybridize with their corresponding substrates, and the risk of leakage would rise. Meanwhile, according to the schematic (Figure 1a) and fluorescence experiments of INH gates (Figure S1), the structure was designed to avoid the risk of leakage, and it was verified that our process of annealing and logic gate construction strategies were simple and effective in leakage suppression.

In terms of the reaction rate of logical operations, due to the different digestion rates between the triple-stranded DNAzymes and the double-stranded DNAzymes, the stable state can be reached within 3 h (Figure S2, curve 4), the reaction rate of which is related to the initial structure and the concentration of the DNAzyme in the system. Here, the longest time in the experiment (Figure S1) was adopted as the reaction time of the calculation circuits to ensure that all reactions reach a stable state. In addition, to avoid sudden variations in fluorescence measurements, all the inputs were added to the solution without oscillation. The reaction rate can be optimized by adjusting the concentration of DNA strands (curve 2 in Figure 5a, and curve 21 °C in Figure S6) or the reaction temperature (Figure S6). However, the most difficult part was to regulate the reaction rates of the system, which promoted the stability and correctness of the results. In future work, the incorporation of highly efficient enzymes such as exonuclease or Nicking enzymes may be considerable in improving system efficiency.

## 5. Conclusions

In this study, DNAzyme was selected as the main processing module, the activity of which was regulated through controlling the assembly of DNAzymes as well as the conformational changes of the secondary structure of its conserved domain. Based on this regulating strategy, a system of DNAzyme assembly was successfully constructed and used to implement logic gates such as the AND gate, OR gate, XOR gate, and INH gate. Furthermore, a modular design was utilized to construct advanced logic circuits. After further cascade, more complex logic circuits were successfully established, such as half-adder, half-subtractor, MUX and DEMUX. In the verification of the logic operation system, the fluorescence experiments with good repeatability and stability were repeated at least three times. In this system, E6 deoxyribozyme was used as the only processing module, and we found that D-F could be utilized to modulate the secondary conformation of the conserved domain. Therefore, in addition to implementing logic operations, the computing system can also implement practical functions by the strategy of threshold control. According to this feature, the concentration of D-F was set as the threshold to implement the operation of the DNA voter with a one-vote veto function. In addition, the system was scalable, and a DNA voter with 2n + 1 inputs can be achieved by increasing the threshold and the types of input to implement large-scale voting. In future work, it is possible to build a highly integrated logic circuits by cascading the basic modules. The proposed strategy helped us to establish a versatile and powerful computing system, and it will serve as an effective method for the construction of highly integrated circuits in the future. It may have broad prospects in biological computing, nano-devices and information processing.

## Data Availability

Not applicable.

## Supplementary Materials

The following supporting information can be downloaded at: https://www.mdpi.com/article/10.3390/biom12040495/s1, Table S1: DNA sequences used in experiment; Figure S1: The fluorescence assay of the INH gate; Figure S2: The fluorescence assay of the OR gate; Figure S3: The implement of the AND gate; Figure S4: The implement of the XOR gate; Figure S5: The PAGE results in the construction of DNAzymes; Figure S6: The reaction rate of DNAzymes under different temperature; Figure S7: The PAGE analysis of the half-adder; Figure S8: The PAGE analysis of the half-subtractor; Figure S9: The PAGE analysis of the 2:1 Multiplexer; Figure S10: The PAGE analysis of the 1:2 Demultiplexer; Figure S11: The hybridization principle and the design of toehold in DNA voter; Figure S12: The fluorescence assay with six groups of different concentration of D-F; Figure S13: The fluorescence values of DNA voter; Figure S14: Design of DNA voter with (2n + 1) inputs.
