# Supplementary material for: Construction of Multiple Logic Circuits Based on Allosteric DNAzymes
Source: Biomolecules. 2022 Mar 24;12(4):495. doi: 10.3390/biom12040495 (PMC9032175; doi:10.3390/biom12040495)
Supplement: Supplementary file 1 [file biomolecules-12-00495-s001.zip › biomolecules-1627000-supplementary.pdf]

Supplementary

Contents

1. DNA sequences used in experiment 2

2. The fluorescence assay of the INH gate 3

3. The fluorescence assay of the OR gate 3

4. The implement of the AND gate 3

5. The implement of the XOR gate 4

6. The PAGE results in the construction of DNAzymes 6

7. The reaction rate of DNAzymes under different temperature 7

8. The PAGE analysis of the half-adder 8

9. The PAGE analysis of the half-subtractor 10

10. The PAGE analysis of the 2:1 Multiplexer 11

11. The PAGE analysis of the 1:2 Demultiplexer 12

12. The hybridization principle and the design of toehold in DNA voter 12

13. The fluorescence assay with six groups of different concentration of D-F 14

14. The fluorescence values of DNA voter 15

15. Design of DNA voter with (2n + 1) inputs 16

1. DNA sequences used in experiment

Table S1. DNA sequences in this study

Name	Sequences (from 5′ to 3′)	Length (n.t.)
**D1$_L$**	TGAAGACAGCGATAGAGATGATGAGATGAGTA	32
**D1$_L$'**	CTTCTTCAGCGATAGAGATGATGAGATGAGTA	32
**D1$_R$**	ATCTCATCATCTCTCACCCATGT TCACTCAGCT	34
**D2$_L$**	CTTCTTCAGCGATACTTAGTGATGATAG	28
**D2$_R$**	AAGTAGTGTATCAGTCACCCATGTTCACTCAGCT	34
**D3$_L$**	CTTCTTCAGCGATACTAATGATACAACA	28
**D3$_R$**	CAATAACCATCCAGTCACCCATGTTCACTCAGCT	34
**TS$_1$**	TTTTTTGAGTGATrAGGTCTTCATTTTTT	28
**TS$_2$**	GAGTGATrAGGAAGAAGTTTTTTTTTTTT	28
**TS$_{L1}$**	TTTTTTGAGTGATA	14
**TS$_{R1}$**	GGTCTTCATTTTTT	14
**TS$_{R2}$**	GGAAGAAGTTTTTTTTTTTT	28
**S$_1$**	ROX-GAGTGATrAGGTCTTCA-BHQ2	16
**S$_2$**	FAM-GAGTGATrAGGAAGAAG-BHQ1	16
**D-F**	AGAGTAGCTGAGTGAACATGGGTGAGAG	28
**D-IN**	CTCTCACCCATGTTCACTCAGCTACTCT	28
**IN$_1$**	TGAAGACAGCGATCTATCATCACTAGATACACTACTT CAATAACCATCCAATGATACAACA	61
**IN$_2$**	TGTTGTATCATTGGATGGTTATTGAAGTAGTGTATCTA GTGATGATAGCACCCATGTTCACTCAGCT	67
**IN$_3$**	CTATCATCACTAGATACACTACTTCAATAACCATCCAA TGATACAACACACCCATGTTCACTCAGCTACTCT	72
**IN$_4$**	TACTCATCTCATCATCTCTATCTGTTGTATCATTGGAT GGTTATTGAAGTAGTGTATCTAGTGATGATAG	70

2. The fluorescence assay of the INH gate

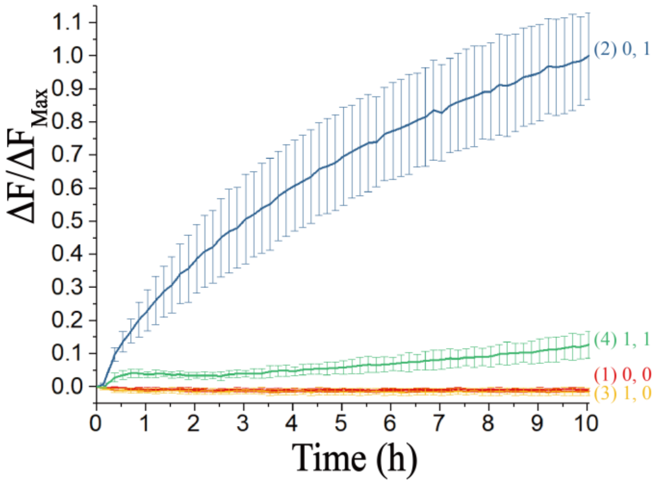

Figure S1. Time-dependent normalized fluorescence curves of the INH gate. (1) No DNA strands are added; (2) DNA strand D-IN is added; (3) DNA strand IN_4 is added; (4) DNA strands D-IN and IN_4 are both added. The fluorescence intensity had a significant increase only in the presence of D-IN. The fluorescence intensities are recorded every 10 min.

3. The fluorescence assay of the OR gate

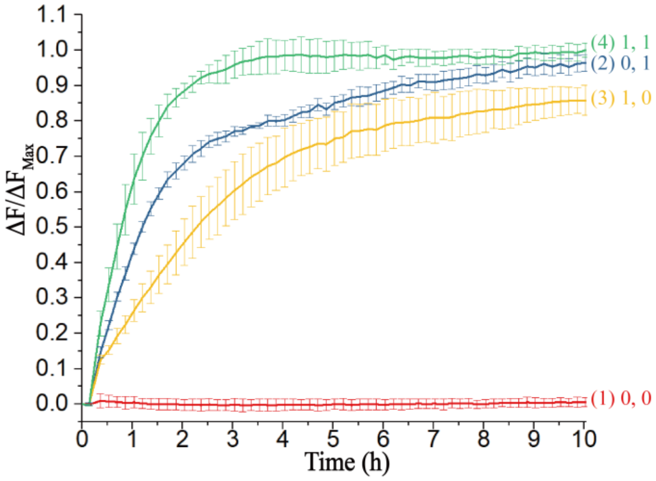

Figure S2. Time-dependent normalized fluorescence curves of the OR gate. (1) No DNA strand is added; (2) DNA strand $D1_R$ is added; (3) DNA strand $D3_R$ is added; (4) DNA strands $D1_R$ and $D3_R$ are both added. The fluorescence intensities are recorded every 10 min.

4. The implement of the AND gate

As shown in Figure S3a, the AND logic gate was implemented by the assembly of the DNAzyme D4. In Figure S3c, only in the co-existence of IN_1 and IN_2, the DNAzyme D4 could be assembled, and the signal of ROX was produced in lane 6. By

comparing lanes 4 and 8, it could be found that part of IN_1 hybridized with TS_1. This is the reason for the band above D4 in lane 6.

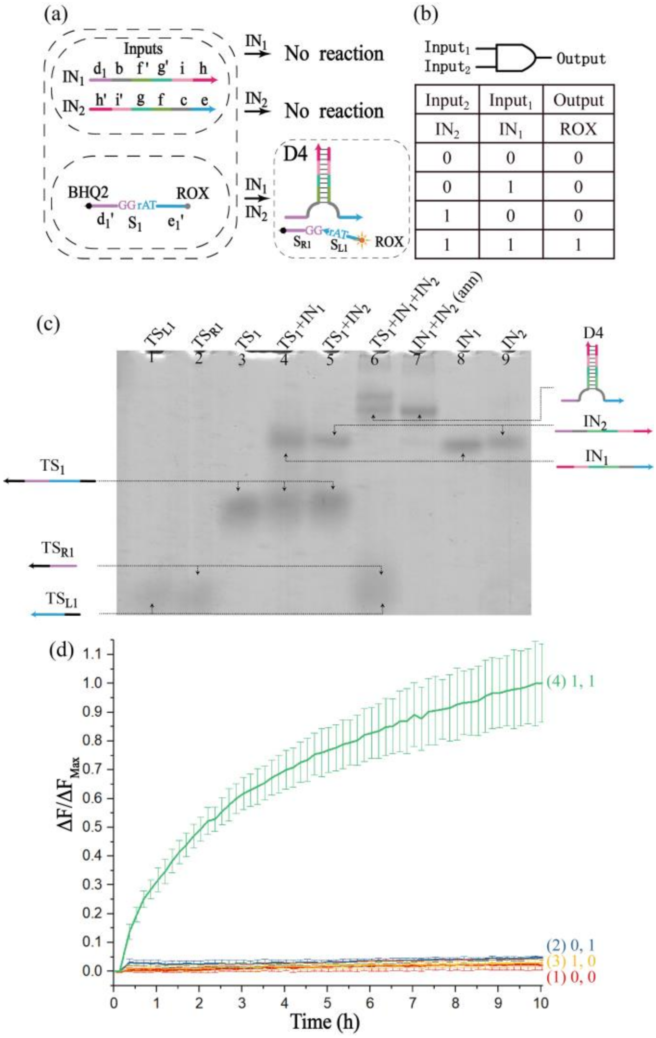

Figure S3. The implementation and verification of the AND gate. (a) Schematic diagram of the AND gate. (b) Truth table and logical operations of the AND gate. (c) PAGE gel of the AND gate. Lanes 1, 2 and 7–9 are for comparison; lanes 3–6 represent the inputs of (0, 0), (0, 1), (1, 0) and (1, 1), respectively. (d) Time-dependent normalized fluorescence curves of ROX. (1) No DNA strand is added; (2) DNA strand IN_1 is added; (3) DNA strand IN_2 is added; (4) DNA strands IN_1 and IN_2 are both added.

5. The implement of the XOR gate

The XOR logic gate implemented by the assembly of D2 and D3 is shown in Figure S4a. The input strand IN_3 had domains f' and g', which were complementary to domain f of $D2_L$ and domain g of $D2_R$, promoting the assembly of D2. IN_4 had domains h' and i', which were complementary to domain h of $D3_L$ and domain i of $D3_R$, promoting the assembly of D3. IN_3 and IN_4 hybridized with each other when they co-existed, then, D2 and D3 would not be assembled.

In Figure S4c, D2 was assembled in lane 7, and it could be verified by Figure S5a. The base numbers of $D2_L$, $D2_R$, $D3_L$ and $D3_R$ are similar, which are 28, 34, 28 and 34nt,

relatively close bands were produced (lanes 3-6). When they were mixed together (lane 9), it could be observed that the bands overlapped, but these 4 DAN strands did not hybridize to each other to produce a double-stranded structure. From lane 10, the substrate TS_2 would hybridize with $D2_L$, $D2_R$, $D3_L$ or $D3_R$. The principle was similar to lane 4 in Figure S3c, but the hybridization process had no effect on the XOR gate. Since the incomplete DNAzyme does not have the cleaving effect, the cleavage product was not appeared in lane 10.

In Figure S4d, D3 was assembled in lane 3 which could be verified by Figure S5b. In lane 4, D3 was activated to digest the substrate TS_2. After the addition of $D2_L$ and $D2_R$ (lane 5), D3 would be assembled as same as lane 4. $D2_L$ and $D2_R$ would not participate in the assembly of D3 (consistent with the lane 10 in Figure S5), and the corresponding bands could be observed (the blue box in lane 5). In lane 7, the hybridized double strand formed by IN_3 and IN_4 could be observed (details in lane 11 of Figure S5a). The addition of substrate TS_2 had no effect on the hybridization of IN_3/IN_4 duplex (lane 8), and the band of TS_2 was free. Lane 6 was the addition of IN_3 on the basis of lane 5, it could be found that the band of D3 did not appear, so the DNAzyme was inactivated and cannot cleave TS_2. Since IN_3 and IN_4 formed a stable double-stranded, the remaining DNA strands included $D2_L$, $D2_R$, $D3_L$, $D3_R$ and TS_2, and the bands in Figure S4d (the red box) were consistent with lane 10 in Figure S4c.

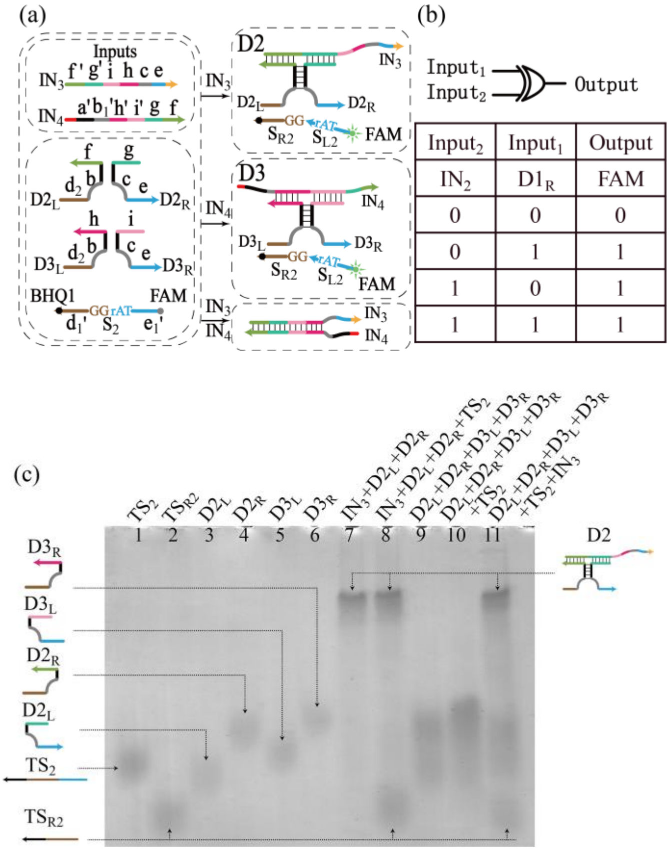

$Input_2$	$Input_1$	Output
IN_2	$D1_R$	FAM
0	0	0
0	1	1
1	0	1
1	1	1

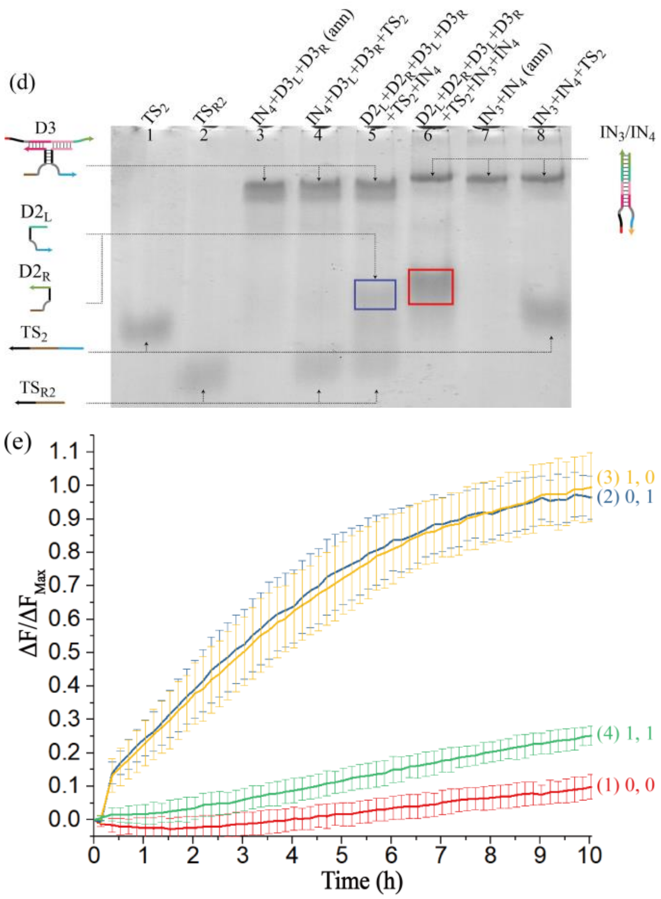

Figure S4. The implementation and verification of the XOR gate. (a) Schematic diagram of the XOR gate. (b) Truth table and logical operations of the XOR gate. (c) PAGE analysis for the inputs of (0, 0) and (0, 1). Lanes 1–9 are for comparison; lanes 10 and 11 represent the inputs of (0, 0) and (0, 1), respectively. (d) PAGE analysis for the inputs of (1, 0) and (1, 1). Lanes 1–4 and lanes 7–8 are for comparison; lanes 5 and 6 represent the inputs of (1, 0) and (1, 1), respectively. (e) Time-dependent normalized fluorescence curves of FAM. (1) No DNA strand is added; (2) DNA strand IN_3 is added; (3) DNA strand IN_4 is added; (4) DNA strands IN_3 and IN_4 are both added.

6. The PAGE results in the construction of DNAzymes

To verify the assembly of D2, D3, D2' and D3', the PAGE gel experiments were carried out, as shown in Figure S5. In Figure S5a, the DNA strand IN_3 could hybridize with $D2_L$ (lane 2) and $D2_R$ (lane 3), respectively. The DNAzyme D2 was further assembled in lane 4, and the same band of D2 without annealing appeared in lane 5 even if there were extra DNA strands $D3_L$ and $D3_R$. Therefore, $D3_L$ and $D3_R$ would not hybridize with IN_3, they would be left after the assembly of D3, and their bands (the red box) appeared in lane 5. Similar to lane 5, the assembly of DNAzyme D3 was verified (lane 10). The hybridization between IN_3 and IN_4 at room temperature (lane 12) was the same with the annealing of IN_3 and IN_4 in lane 11.

In Figure S5b, the DNA strands IN_3 and IN_4 were replaced with IN_1 and IN_2, so, the same hybridization principle was used to verify the assembly of the DNAzymes D2'

and D3', the bands of which were observed in lane 5 and lane 10. The hybridization between IN_1 and IN_2 (lane 12) was the same with the structure after the annealing of IN_1 and IN_2.

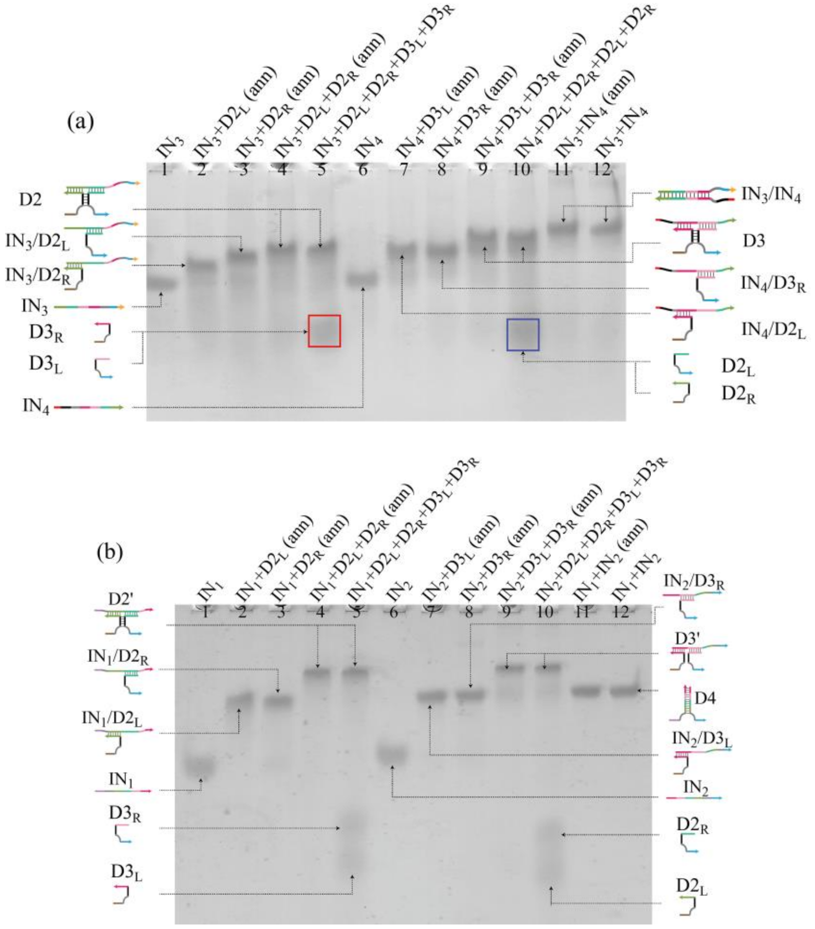

Figure S5. PAGE analysis for the assembly of triple-stranded DNAzymes. (a) PAGE gel analysis for the assembly of triple-stranded DNAzymes D2 and D3. (b) PAGE gel analysis for the assembly of triple-stranded DNAzymes D2' and D3'.

7. The reaction rate of DNAzymes under different temperature

Four groups of different temperatures were introduced, which were 21 °C, 24 °C, 27 °C and 30 °C, respectively. In Figures S6a and S6b, as the temperature increased, the reaction rate of D1' and D2 decreased significantly, and the final fluorescence value decreased, but D4 was the opposite (Figure S6c). It is possible that the increase in temperature is conducive to the movement of DNA strands, leading to the acceleration of biochemical reactions. Therefore, the reaction rate of D4 (48bp) with a stable double-stranded structure (Figure S6d) was promoted when the temperature was increased. But D1' (14bp) and D2 (assembled by three DNA strands) were sensitive to temperature, and the increase in temperature reduced the stability of their structure, causing a decrease in the reaction rate.

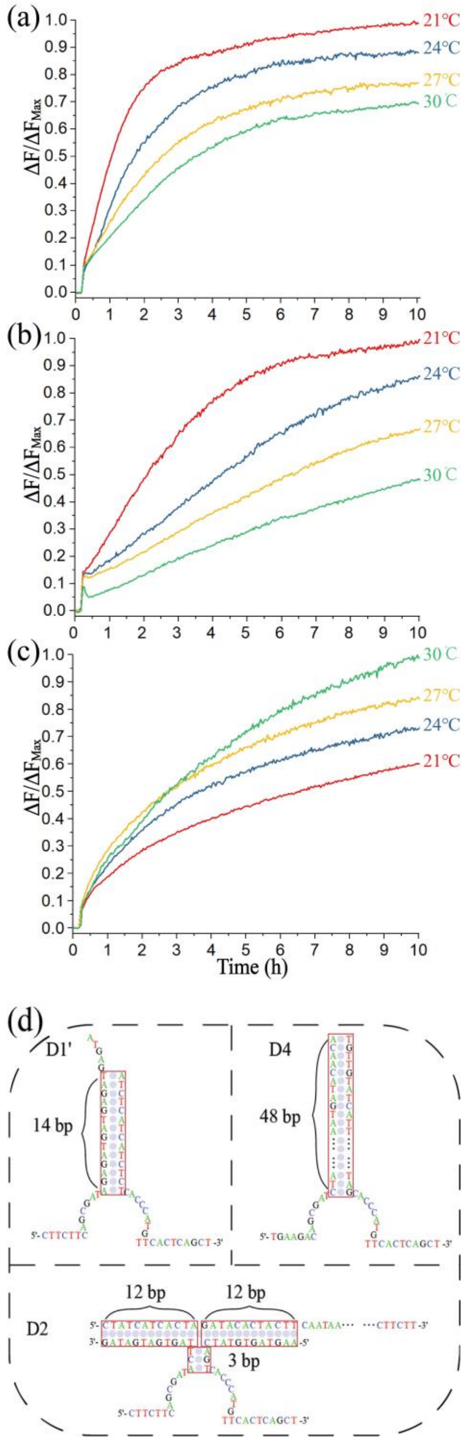

Figure S6. Time-dependent normalized fluorescence curves under different temperature. (a) The reaction rate of assembled D1'. (b) The reaction rate of assembled D2. (c) The reaction rate of assembled D4. (d) Structures of assembled D1', D4 and D2.

8. The PAGE analysis of the half-adder

In Figure S7a, a band of D2' for comparison was set in lane 4 (consistent with lane 4 in Figure S5b). With the addition of TS_2 (lane 5), the activated D2' would cleave TS_2,

and the band of TS_{R2} could be observed. Lane 6 was the same with lane 9 (Figure S4c). Since $D2_L$, $D2_R$, $D3_L$ and $D3_R$ contained the complementary domains that can hybridize with TS_1 and TS_2, similar to lane 10 in Figure S4c, the bands in lane 7 (the blue box) would be higher than that in lane 6 (the red box), but the hybridization would not cause the cleavage of TS_1 or TS_2 (no products appeared). After the addition of IN_1, the band of D2' appeared in lane 8, so, TS_2 was cleaved, but TS_1 could not be digested. The bands (the yellow box) contained $D2_L$, $D2_R$, $D3_L$, $D3_R$ and TS1, were higher than the red box in lane 6.

In Figure S7b, similar to lane 8 in Figure S7a, the assembled D3' cleaved TS_2 in lane 6, but it cannot digest TS_1 (there was no TS_{L1} and TS_{L2}), so the left DNA strands included $D2_L$, $D2_R$ and TS_1 (the red box). In lane 7, only IN_1/IN_2 was assembled by the hybridization of IN_1 and IN_2, and neither D2' nor D3' was assembled. So, TS_2 cannot be cleaved (there was no substrate corresponding to TS_2), but TS1 would be digested by IN_1/IN_2 (the bands of TS_{L1} and TS_{R1} could be observed). The left DNA strands included $D2_L$, $D2_R$, $D3_L$, $D3_R$ and TS_2 (the yellow box), which was the same with lane 10 in Figure S4c.

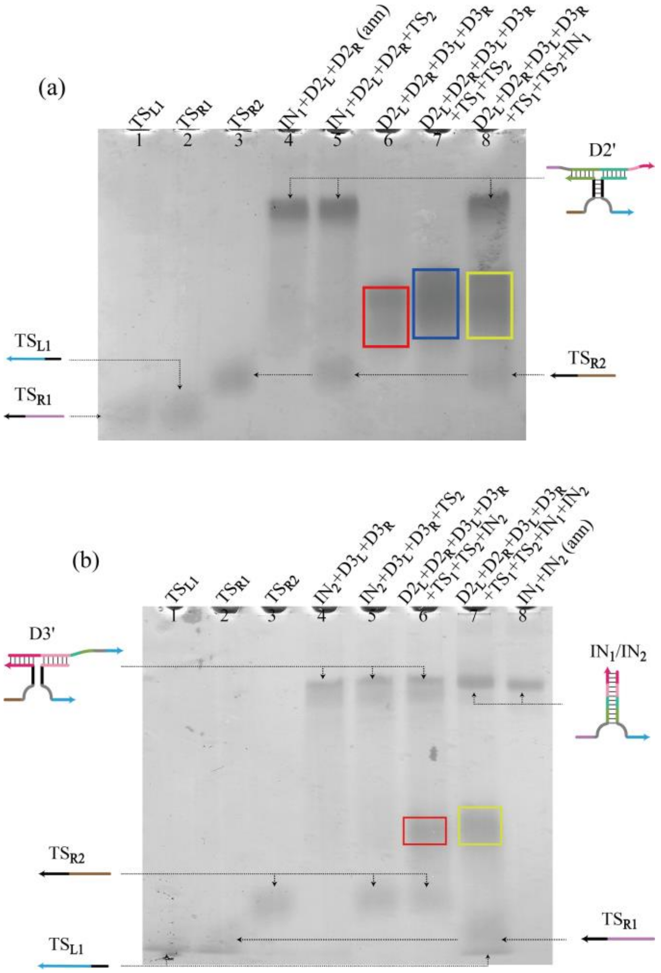

Figure S7. PAGE gels of the half-adder. (a) Lanes 1–6 are for comparison; lanes 7 and 8 represent the inputs of (0, 0), (0, 1), respectively. (b) Lanes 1–5 and lane 8 are for comparison; lanes 6 and 7 represent the inputs of (1, 0), (1, 1), respectively.

9. The PAGE analysis of the half-subtractor

Since a large number of DNA strands were added in the half-subtractor, according to the above experimental results, it can be derived that the cleavage of the substrates was related to the assembly of the DNAzyme, and the single strand of DNA would not digest any substrate, but the bands of single strand may overlap each other (Figure S7a, lane 7). Therefore, in order to avoid the influence of single-strand overlap on the experimental analysis, the stable synthesis of each structure in the reaction would be explored in the following works, which was the verification of the successful operation of the logic circuits.

In Figure S8a, when there was no input in lane 5, no DNAzyme were activated (no DNAzyme band). When IN_3 was added in lane 6, D1, D2 and D2/D-F were stably formed in the solution, which could digest TS_1 and TS_2.

In Figure S8b, with the addition of IN_4 (lane 4), D3 was assembled, and the band of $D1_R$/D-F could be observed. With the addition of IN_3 and IN_4 (lane 5), IN_3 and IN_4 would hybridize preferentially to form a double strand, and D2 and D3 cannot be activated (there was no band of DNAzyme). In addition, in the actual reaction process, after the addition of IN_3 and IN_4, $D1_L$ and D-F cannot simultaneously hybridize with IN_3/IN_4 (the band of IN_3/IN_4/D-F/$D1_L$ did not appear).

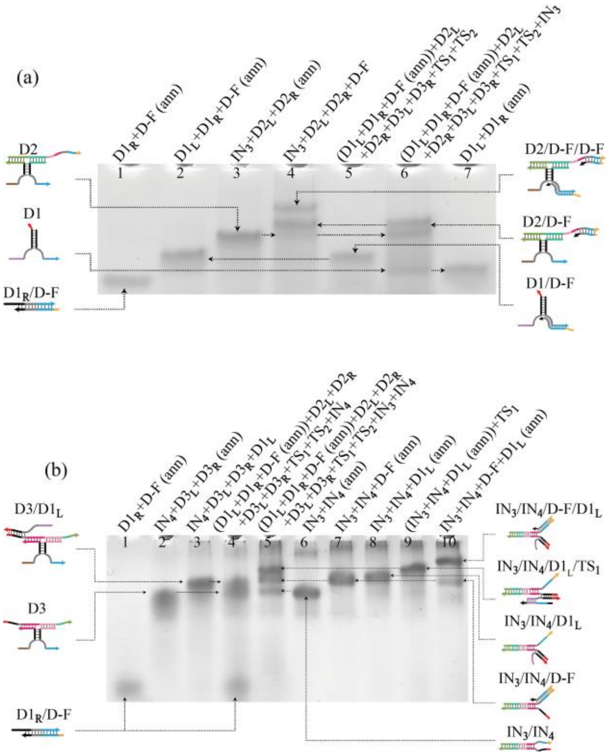

Figure S8. PAGE gels of the half-subtractor. (a) Lanes 1–4 and lane 7 are for comparison; lanes 5 and 6 represent the inputs of (0, 0), (0, 1), respectively. (b) Lanes 1–3 and 6–10 are for the comparison; lanes 4 and 5 represent the inputs of (1, 0), (1, 1), respectively.

10. The PAGE analysis of the 2:1 Multiplexer

In Figure S9a, when no input was added (lane 8), $D1_L'$, $D1_R$ and D-F would form D1'/D-F, and the band of D1'/D-F was consistent with lane 4. The left DNA strands were $D3_L$ and TS_2. Since $D3_L$ would hybridize with TS_2, the red box will be higher than the band in lane 1 (blue box), but D1' cannot be activated (where was no band of D1'). Similarly, the addition of $D3_R$ (lane 10) will also hybridize with TS_2 (the yellow box), and the band was higher than the band in lane 2 (the blue box). After adding D-IN in lane 9, D1' was activated, and the band of D-IN/D-F could be observed. When D-IN and $D3_R$ were added, the bands of D1' and D-IN/D-F would appear in lane 11, while $D3_L$ and $D3_R$ were left (the blue box).

In Figure S9b, when IN_4 was added in lane 6, the band of D1'/D-F disappeared while the band of $D1_R$/D-F could be observed, and no DNAzyme was assembled. When IN_4 and D-IN were added (lane 7), it was similar to lane 6, but the band of $D1_R$/D-F disappeared and the band of D-IN/D-F could be observed in the lane 7. When IN_4 and $D3_R$ were added (lane 8), D3 was successfully assembled (the band of $D3/D1_L'$), and there was no band of D1' in the lane 8. When all inputs were added to the system (lane 9), the bands of $D3/D1_L'$, $IN_4/D3_L/D1_L'$ and D-IN/D-F appeared, so, DNAzyme D3 was successfully assembled.

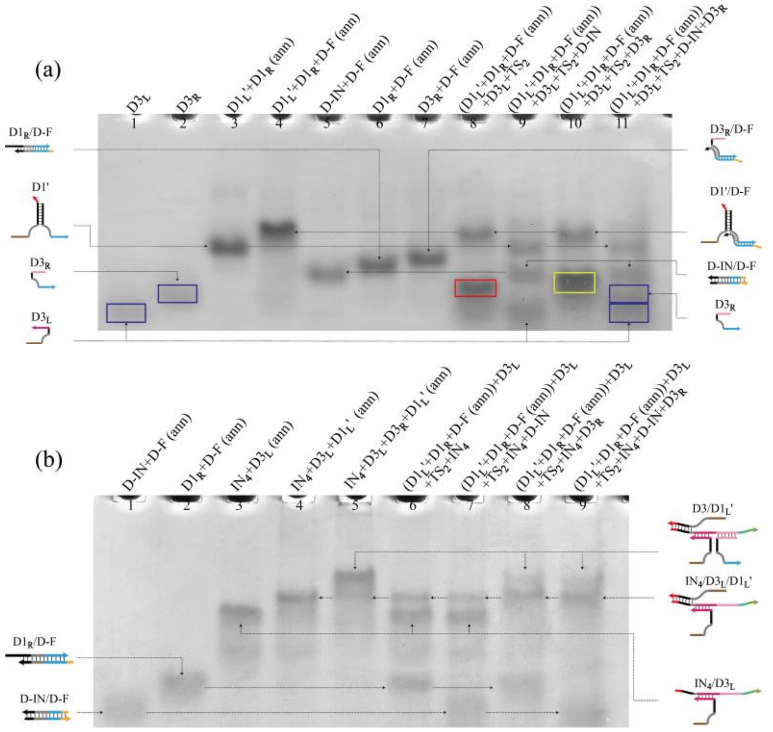

Figure S9. PAGE gels of the 2:1 MUX. (a) Lanes 1–7 are for comparison; lanes 8–11 represent the inputs of (0, 0, 0), (0, 0, 1), (0, 1, 0) and (0, 1, 1), respectively. (b) Lanes 1-5 are for the comparison; lanes 6-9 represent the inputs of (1, 0, 0), (1, 0, 1), (1, 1, 0) and (1, 1, 1), respectively.

11. The PAGE analysis of the 1:2 Demultiplexer

The assembly of D3' in lane 4 was consistent with lane 9 in Figure S5b, from lanes 7, 8 and 9 of Figure S5b, it can be found that IN_2, $D3_L$, and $D3_R$ cannot completely form D3'. So, there were the bands of $IN_2/D3_L$ and $IN_2/D3_R$, and two bands appeared in lanes 4 and 6 (corresponding to lane 9 in Figure S5b). When no inputs were added, there was no band of DNAzyme assembled in lane 5. When IN_1 was added, due to the presence of TS_1 in the solution, IN_1 would hybridize with TS_1 in lane 7, which was confirmed by lanes 4 and 8 in Figure S3c. In lane 8, when IN_1 and IN_2 were added, they would hybridize with each other preferentially, and D3' cannot be assembled.

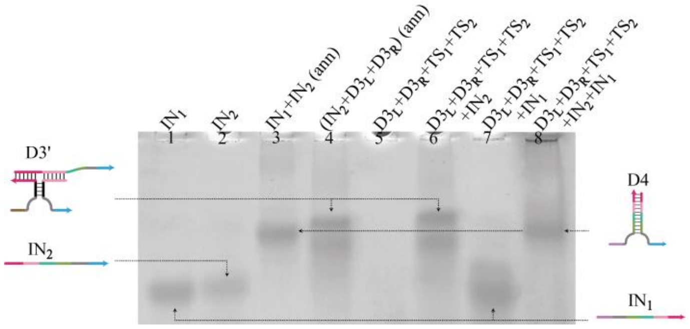

Figure S10. PAGE gel of the 1:2 DEMUX. Lanes 1–4 are for comparison; lanes 5–8 represent the inputs of (0, 0), (0, 1), (1, 0) and (1, 1), respectively.

12. The hybridization principle and the design of toehold in DNA voter

As shown in Figure S11a. These three inputs could hybridize with D-F to form a stable double-strand due to the relatively long hybridization regions (28 bp, 23 bp and 19 bp), which were verified in lanes 4, 6 (Figure S11d) and in lane 5 (Figure S11e). And the double-strand formed by the hybridization of inputs and S_2 was not stable due to the short hybridization region (6 bp). According to the above, the input strands had a better affinity with D-F relative to S_2, and the hybridization between inputs and D-F was irreversible, because there was no toehold for S_2 to displace D-F. So, at the presence of D-F, strand S_2 would not hybridize with any DNAzymes, which inhibited the digesting reaction, as shown in Figure S11b. In Figure S11b, DNA strand D-F hybridized with the three inputs to change the secondary structure of DNAzyme. DNA strand D-IN, one of the three inputs, had the priority over the other two inputs, and it was designed as completely complementary to D-F.

In Figure S11c, DNA strand D-IN displaces $D1_R$ and $D2_R$ from D-F to activate the DNAzymes based on the toehold mediated strand displacement reaction. Even if other two inputs hybridized with D-F, D-IN would displace them from D-F, trigged by the exposed yellow region (toehold), and the length of the toehold was set as 5 bases.

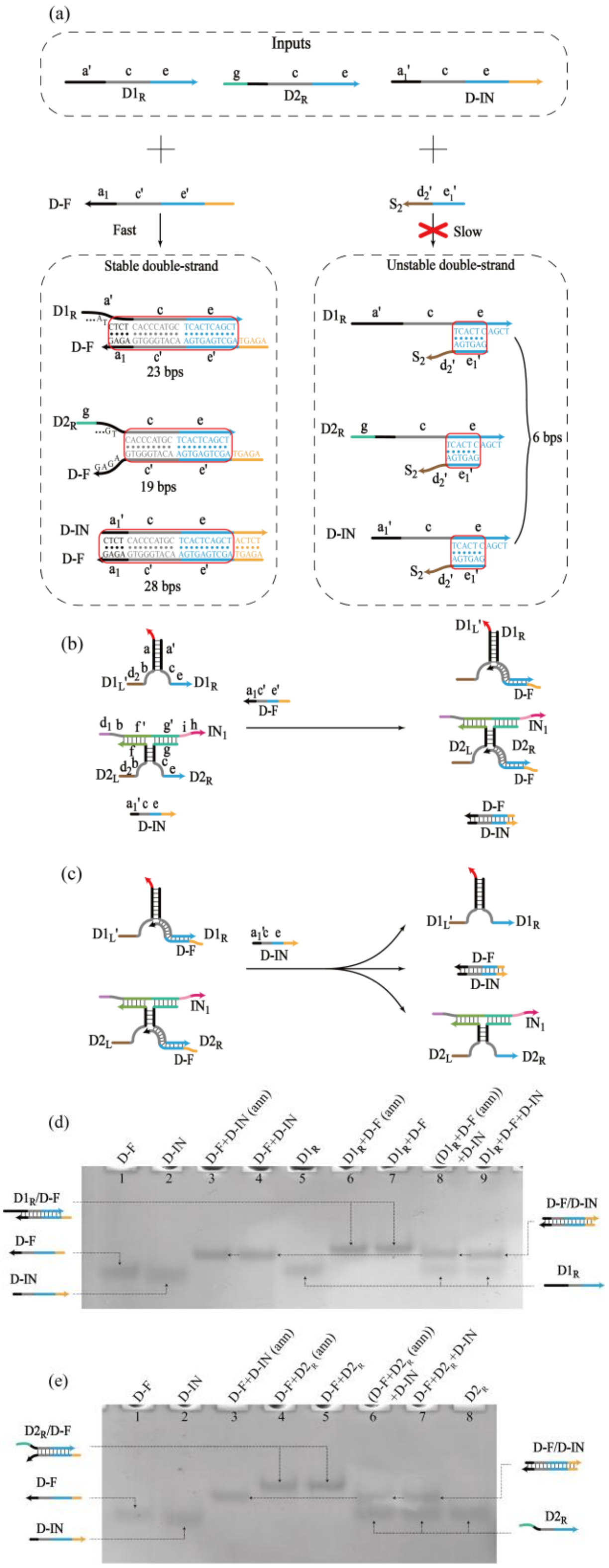

Figure S11. The hybridization between inputs and D-F. (a) The design of toehold in each strand. (b) Schematic diagram of the hybridization principle between inputs and D-F. (c) DNA strand D-IN displaces $D1_R$ and $D2_R$ from D-F to activate the DNAzymes based on the toehold mediated strand displacement reaction. (d) PAGE gel to verify that D-IN displaces $D1_R$ from D-F. (e) PAGE gel to verify that D-IN displaces $D2_R$ from D-F.

13. The fluorescence assay with six groups of different concentration of D-F

In Figure S12a, the final fluorescence with the addition of $D1_R$ (D-F was 0.9× in Figure 8a) was set as the maximum leakage, and the fluorescence curves with the addition of $D2_R$ were normalized. Compared with the fluorescence curves in Figure 8a, the reaction rate of DNAzyme in Figure S12a was slow, and the difference in the structure of DNAzyme was the main reason. D1' was assembled by two DNA strands, while D2' was assembled by three DNA strands, which leaded to the differences in their rate of assembly and reaction. As shown in Figures S6a and S6b, at 21 °C, the curve in S6a reached equilibrium first, while the reaction rate of S6b was slow. Therefore, the digestion rate of D2' was slower than that of D1'. In Figure 8a, the fluorescence was negative when the concentration of D-F was greater than 1×. But the corresponding leakage was actually produced (curves 2, 3, 4, 5 and 6 in Figure 8a). Since the hybridization between DNA strands was parallel, $D1_R$ not only hybridized with D-F in the presence of $D1_R$, but also hybridized with $D1_L$' to generate activated D1', which also directly caused the rise in fluorescence. When D-F was 0.9×, the amount of D-F was less than $D1_R$, part of D1' was activated to continuously digest substrate. Since the reaction of activated double-stranded DNAzyme with the substrate was rapid (Figure S6a), so the leakage of curve 1 in Figure 8a was obvious.

Similarly, when $D2_R$ was added to the solution, the leakage was also accompanied. Due to the slow digestion rate of D2', the leakage was difficult to be observed (curves 2, 3, 4, 5 and 6 in Figure S12a). However, in curve 1 of Figure S12, due to the assembly of activated D2', there would be a leakage in fluorescence, and the rate was slower than that of curve 1 in Figure 8a. In addition, the reaction rate in Figure 8b (the addition of D-IN and $D2_R$) was slower than that in Figure S12b (the addition of D-IN and $D1_R$).

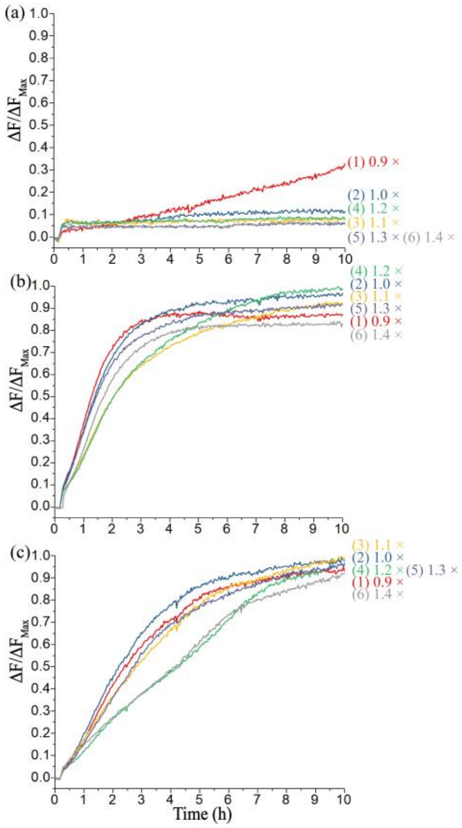

Figure S12. Fluorescence analysis for different concentrations of DNA strand D-F, which are 0.9×, 1.0×, 1.1×, 1.2×, 1.3× and 1.4× 0.5 μM, respectively. (a) DNA strand $D2_R$ is added; (b) DNA strands D-IN and $D1_R$ are added; (c) DNA strands $D1_R$ and $D2_R$ are added.

14. The fluorescence values of DNA voter

In Figure S13, the initial (the red column) and final fluorescence value (the blue column) of results are shown in Figure S13.

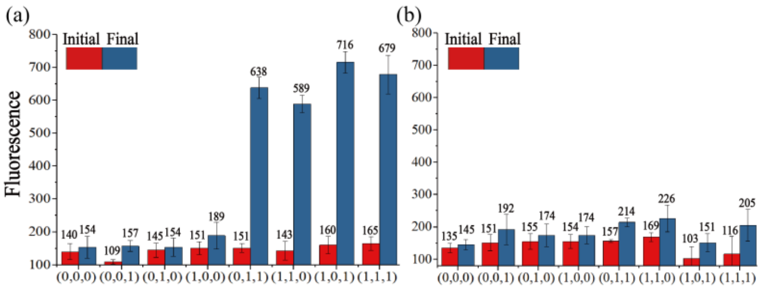

Figure S13. The fluorescence changes of output with (a) / without (b) the veto vote.

15. Design of DNA voter with (2n + 1) inputs

In Figure S14, DNAzymes were inactivated in the presence of less than half inputs, but DNAzymes were activated with the addition of more than half inputs by increasing the threshold and the types of inputs.

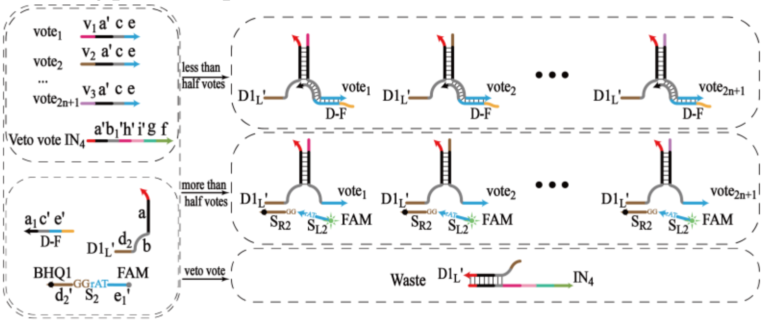

Veto-vote	Vote$_{2n+1}$	…	Vote$_2$	Vote$_1$	Result
0	Less than half votes				Reject
	More than half votes				Approve
1	0/1	0/1	0/1	0/1	Reject

Figure S14. Schematic diagram of the 2n + 1 (n=1, 2, 3…) inputs DNA voter with one-vote veto function and its truth table.
